# The Multum in Parvo Reference and Dose Book

**Published:** 1879-11

**Authors:** 


THE MULTUM IN PARVO REFERENCE AND DOSE BOOK. By C. Henri Leonard, M.A., M.D. Third Edition Revised and Enlarged—23rd Thousand. 1879. Published by the Author.

A neat little reference book, with many items of useful information.

